# Making decisions about treatment for young people diagnosed with depressive disorders: a qualitative study of clinicians’ experiences

**DOI:** 10.1186/1471-244X-13-335

**Published:** 2013-12-12

**Authors:** Magenta B Simmons, Sarah E Hetrick, Anthony F Jorm

**Affiliations:** 1 headspace Centre of Excellence in Youth Mental Health, Orygen Youth Health Research Centre, Centre for Youth Mental Health, The University of Melbourne, Locked Bag 10, Parkville 3052, Victoria, Australia; 2 Population Mental Health Group, Melbourne School of Population Health, The University of Melbourne, Parkville, Victoria 3010, Australia; 3 Orygen Youth Health Research Centre, Centre for Youth Mental Health, The University of Melbourne, Locked Bag 10, Parkville 3052, Victoria, Australia

## Abstract

**Background:**

The imperative to provide effective treatment for young people diagnosed with depressive disorders is complicated by several factors including the unclear effectiveness of treatment options. Within this context, little is known about how treatment decisions are made for this population.

**Methods:**

In order to explore the experiences and beliefs of clinicians about treatment decision making for this population, semi-structured, qualitative interviews were conducted with 22 psychiatrists, general practitioners and allied health professionals from health care settings including specialist mental health services and primary health care. Interviews were audio taped, transcribed verbatim and analysed using thematic analysis.

**Results:**

Clinicians largely reported and endorsed a collaborative model of treatment decision making for youth depression, although several exceptions to this approach were also described (e.g. when risk issues were present), highlighting a need to adapt the decision-making style to the characteristics and needs of the client. A differentiation was made between the decision-making processes (e.g. sharing of information) and who makes the decision. Caregiver involvement was seen as optional, especially in situations where no caregivers were involved, but ideal and useful if the caregivers were supportive. Gaps between the type and amount of information clinicians wanted to give their clients and what they actually gave them were reported (e.g. having fact sheets on hand). A broad range of barriers to involving clients and caregivers in decision-making processes were described relating to four levels (client and caregiver, clinician, service and broader levels) and suggestions were given to help overcome these barriers, including up-to-date, accessible and relevant information.

**Conclusions:**

The current data support a collaborative model of treatment decision making for youth depression which: 1) focuses on the decision-making processes rather than who actually makes the decision; 2) is flexible to the individual needs and characteristics of the client; and 3) where caregiver involvement is optional. Shared decision making interventions and the use of decision aids should be considered for this area.

## Background

Depression is a highly prevalent mental disorder that can result in negative outcomes in several domains, including social, occupational, physical and emotional functioning [1,2]. Adolescence is a significant time for both the onset of depression and the importance of providing treatment. By the age of 18 years, one in every five people will have experienced depression [3]. Providing effective treatment in a timely manner is crucial [4] and can serve several purposes: to relieve the current episode, to prevent further episodes, and to establish positive treatment experiences, maximising the potential of seeking help in the future. The provision of treatment, however, is complicated by issues such as difficulties with engagement [5], access to treatment and services [6-8] and delays to accessing treatment [9,10], which may be in turn be influenced by barriers such as stigma [11] and negative attitudes to treatment [12].

These factors mean that treatment decision making for this population is complex and challenging. Guidelines for the treatment of moderate-to-severe depression in children and adolescents (e.g. [13]) recommend an initial trial of psychological therapy (e.g. cognitive behavioural therapy (CBT); interpersonal therapy (IPT)) followed by the addition of antidepressant medication if this is unsuccessful. A recent meta-analysis of CBT for adolescent depression demonstrated a decrease in effect size over time [14]. Despite large effect sizes in earlier trials, newer studies with more rigorous methodologies have not had the same success. Other likely factors influencing effect size include severity of depression and comorbidity of participants [15]. IPT has demonstrated effectiveness, but only a small number of studies have been conducted [16] and the provision of both CBT and IPT depends on the availability of trained therapists. In terms of antidepressant medication, a recent Cochrane review found that although there was a statistically significant effect in favour of medication compared with placebo, the difference was only small and the clinical relevance of this reduction is not known [17]. Additionally, the potential relationship between SSRIs (and new generation antidepressants) and an increase in suicidal thinking and behaviours has raised safety concerns for this population [18], and there has been controversy in general surrounding both the effectiveness and potential risks of such medications [19-21].

Given these complexities, treatment decision making in this area remains a challenge for health services and professionals. A study investigating treatment decision making experiences of adults diagnosed with depressive disorders suggested that the context for such treatment decisions was different to decisions in other health areas, due to factors such as perceptions of depression including stigma about the disorder; delays in seeking help; and the lack of information given to clients about depression and treatment options [22]. This is likely to be even more the case for children and adolescents diagnosed with depressive disorders due to the uncertainty of treatment options. Little is known, however, about the ways in which decisions are made about treatment for young people diagnosed with depression. Two recent reviews of studies investigating shared decision making for mental disorders, for example, yielded no results for studies with young people [23,24].

We have previously reported data on the experiences and beliefs of young people who had been diagnosed with depressive disorders and caregivers of such young people [25]. In semi-structured qualitative interviews with ten young people and five caregivers from a specialist mental health service and primary care, we found that experiences of involvement in treatment decision making for clients varied and were influenced by their own experiences, the clinicians treating them and the service settings. Most young people wanted to be involved to some degree, however this varied across clients and within clients over time. Experiences for caregivers were more homogenous in that they tended to make practical contributions rather than feel truly involved. Several barriers to being involved were reported by both clients and caregivers, covering issues at the client-level (e.g. age); clinician-level (e.g. trust with clinician); service-level (e.g. waiting lists and length of appointment times); and broader barriers (e.g. stigma). Overall, this study suggested that these barriers could be overcome to some extent by explicitly offering involvement to young people diagnosed with depressive disorders when it comes to making decisions about their own treatment, and that caregivers should be involved where appropriate, but at a minimum they should be provided with quality psychoeducational materials.

The present study complements this earlier research by considering the experiences and beliefs of clinicians from a range of health services, including primary health care, enhanced primary health care, child and adolescent mental health services and specialist mental health services. Due to the paucity of data on this topic, this study aimed to provide a descriptive account of the ways in which clinicians reported making treatment decisions, their beliefs about how decisions should be made and barriers to making good treatment decisions.

## Method

### Research team and reflexivity

The interviews were conducted by MS, a female PhD candidate with experience in conducting qualitative, semi-structured and structured clinical research interviews with young people diagnosed with mental disorders. A relationship was established briefly with each interviewee by telephone and again in person before the interview.

### Study design

This project was designed within a social constructionist epistemology in that the interview schedule was designed to ‘lead’ the interviewee as little as possible, and the dialogue between the interviewer and interviewee was treated as equally relevant to the data. Within this framework, thematic analysis was employed as the methodological approach [26]. The interview schedule (see below) was adapted from a previously published focus group schedule [27] in order to meet the aims and context of the project. The interview schedule was revised after the first 10 interviews in order to cover topics raised in earlier interviews (see notes below). Ethics approval was obtained from the relevant local committee (Melbourne Health Research and Ethics Committee; reference number 2008.19) and written, informed consent was obtained from participants.

### Interview schedule

#### **Experiences**

•What different types of experiences (e.g. different clients you’ve seen, different services you have worked at) have you had?

•What types of decisions have you made/your clients made about treatment options?

•What options do your clients have?

•How do you present these options?

•How involved were you in making these decisions?

•How involved were your clients? Caregivers?

•Do you inform your clients/their caregivers of the possible risks and benefits of each treatment option?

•Have you ever disagreed about a treatment decision?

#### **Beliefs**

•Do you wish the decision-making process was different?

•If yes, how so?

•How important is everyone’s input into the decision-making process?

•How important are client, caregiver and clinician values?

•Who should weigh up the risks?

•Pros/cons of clients/caregivers being involved in the decision-making process?

•Any barriers (e.g. in the system) to being involved?

•Anything that could improve decision-making process?

•What constitutes *true* involvement for you?

### Participant selection

A purposive sample was recruited and interviews were conducted until a range of professionals with a range of experiences and views had been covered. The project was presented to clinicians at staff and clinical review meetings. Twenty-two clinicians participated in total.

### Inclusion criteria

Clinicians who had provided treatment to young people aged 12–18 years old for a major depressive disorder (MDD).

### Setting

Participants were recruited from two main services: Orygen Youth Health (OYH) (a specialist youth mental health service for young people aged 15–24 living in the north western metropolitan area of Melbourne, Australia) and headspace Barwon (an enhanced general practice service for young people aged 12–25 living in the satellite city of Geelong, 75 kms south-west of Melbourne). Treatment at OYH involves (after an initial assessment period) a multidisciplinary treating team including an allied health professional acting as a case manager and a psychiatrist as required. Case management and psychological therapy are a core part of treatment, so treatment decisions (other than whether or not to engage with the service) include those related to medication, admission to inpatient services, and additional psychosocial services such as group programs and vocational assistance. Treatment at headspace Barwon usually involves an initial assessment, then allocation to a range of healthcare professionals, mainly psychologists working in a private practice model. Clients can also see a general practitioner (GP) at any time. Treatment decisions therefore include whether or not to engage in psychological therapy, take medication, or engage in a range of other psychosocial interventions. As clinicians work together at both services, it is possible that two or more clinicians are involved in treatment decisions (e.g. psychologist attending a medical review to discuss medication). Additional participants were recruited from associated mental and general health services.

### Data collection

The interviews were audio recorded and transcribed using an orthographic (verbatim) style, and field notes were taken during each interview. Transcripts were edited to preserve the anonymity of participants (e.g. names removed and replaced with “[name of clinician]”). Each interview lasted between 22 and 44 minutes (mean = 35.52 minutes).

### Data analysis

The interview schedule was based on broad themes relating to decision making processes, however new themes were also derived from the data. Due to the paucity of data on this topic, the analysis was designed to be descriptive in nature. Presentations were given at the two main services and data were summarized in a report sent to participants inviting feedback. No feedback necessitating changes to the analysis was received.

A random sample of 12 transcriptions was identified (using SPSS) and checked for accuracy by an independent person, however no significant errors were found. Initial coding was undertaken during the transcription process, partly in order to inform the decision about whether or not thematic saturation had been reached. Based on this, overarching themes were established and transcripts were then recoded once the overall thematic map was drafted. One author (MS) undertook the main analysis and during stages 4 (reviewing themes) and 5 (defining and naming themes) of the thematic analysis process [26] the analysis and relevant audit trail for each section were discussed with the co-authors (SH and AJ). Critical appraisals of the analysis were provided and transcripts were checked where necessary. No software package was used for analysis.

## Results

The key findings are summarized in Table 1. A more detailed description is given below.

**Table 1:** Summary of results from clinicians related to experiences, beliefs and barriers to involvement

**Theme**	**Findings**
**Approach to treatment decision making**	
*Decision-making model*	• Vast majority of clinicians employ a collaborative approach to decision-making processes either some, or all, of the time
	• Ultimate decision rests with the client, but clinicians have professional responsibilities
*Who should weigh up the potential risks and benefits of different treatment options?*	• Clinicians present treatment options to clients and discuss the potential risks and benefits of treatment options
	• Most clinicians support a collaborative approach to considering potential risks and benefits of treatment options
	• Small number of clinicians felt that either they should do it themselves or that clients should do it with their support
	• Clinicians role in weighing up risks and benefits ranged from supportive to directive, and included provision of information as a key task
	• Some clinicians made a distinction about the decision-making process and who actually makes the decision
*Client values and preferences*	• Values and preferences important part of treatment decision making, including cultural and religious values, and relevant individual characteristics
	• Clinicians have opinions about the merits of different treatment options and explain the rationale for their choice to clients, particularly when disagreements arise
	• Clinicians make some decisions before being discussed with clients
*Asking explicitly about preference for involvement*	• None of the clinicians ask clients explicitly about their preferred level of involvement in treatment decision making
**Exceptions to decision making approaches taken by clinicians**	• Four main circumstances leading to a more paternalistic style of treatment decision making: depression severity and associated decline in functioning; perceived risk levels (i.e. to risk to self or others); perceived client preference for involvement; age/developmental stage of the client
	• These situations involved a shift in dynamics rather than employing a strictly paternalistic approach
	• Several clinicians felt that the client should still have the final decision unless they were being treated involuntarily
	• Caregiver involvement necessary for younger clients
**Reasons for involving clients**	• Therapeutic in and of itself
	• To facilitate engagement of the client
	• The “right thing to do”
	• Developmental stage/age
	• To help young people develop a sense of autonomy
	• “Higher success rate” with treatment
	• Affording clients a “sense of control”
	• Adherence and therefore longer lasting benefits of treatment
	• To promote future help seeking
**Caregiver involvement**	• Optional and based on the preference of the client
	• Encouraged but not mandatory
	• Policy at some services to never insist on caregiver involvement
	• “Ideal” or “essential”; but only with client consent
	• More or less caregiver involvement based on age/maturity of client; depression severity and risk issues; capacity to make decisions
	• Some clients do not have caregivers
	• Usually involves practical assistance and provision of collateral information rather than sharing decision
	• Providing information to caregivers seen as important
	• Potential negative outcomes
**Conceptualising involvement**	
*What constitutes true involvement?*	• “Joint understanding”
	• Engagement
	• Insight
	• Willingness to be there
	• Having an opinion; feeling comfortable to openly criticise experiences of treatment
	• Freedom for “mutual agreement and disagreement”
	• “Two way conversation”
	• “Equal conversation”
	• Respect for choices
	• Competency
	• Comprehension
	• Level of articulateness
**Information provision**	
*General*	• Topics typically covered (e.g. depression, therapy, medication)
	• Information sourcing and provision (e.g. fact sheets, websites)
	• Reasons for varying the content or format of information (e.g. younger clients)
*Describing potential risks and benefits of treatment options*	• Potential benefits of CBT: effectiveness in general and in terms of relapse prevention; that it can be tailored to the client
	• Potential risks of CBT: disengaging from therapy; poor connection with therapist; feeling worse before feeling better; gaining insight may cause distress
	• Potential benefits of medication: Likely to help faster than psychological therapy and might help to do therapy but would not “cure anything”; not a “magic bullet”; would not work straight away; evidence favours combination of CBT and medication
	• Potential risks of medication: important to discuss to avoid non-adherence, so clients could monitor seek treatment for side effects, and because it’s a clinician’s duty of care; different levels of information provided; increased risk of suicidality
*Tailoring information*	• Information simplified for younger clients; those with lower levels of comprehension/literacy skills or cognitive impairment
	• Information provision varied according to clinician
*Information formats*	• Information mostly conveyed orally
	• Some clinicians felt that written information was useful; others did not; some felt web based tools helped engage young people
	• Psychologists assumed psychiatrists used fact sheets; psychiatrists did not report consistent use of fact sheets
**Negative aspects of client involvement**	• Few negative aspects reported
	• If client decided not to engage in, or disengage from, treatment; if a client did not comprehend/process information sufficient to make a decision; if the family does not support the young person’s decision and this causes conflict or stress; potential burden
**Disagreements**	
*Disagreements with clients*	• Some clinicians reported no disagreements; others reported minor disagreements (e.g. “little bumps”); others reported more significant disagreements (e.g. non-attendance)
	• Responses to disagreements included “actively exploring” reasons and/or unresolved questions; presentation and/or representation of information and/or clinician rationale
	• Ultimately up to client
*Disagreements with caregivers*	• Majority involved caregivers either wanting, not wanting, or not being told about medication prescribed to clients
	• Responses to disagreements included involving caregivers earlier in the process; further exploring and understanding the perspective of the caregiver; and restating the rationale or justification for their position
**Barriers and facilitators to involving clients and caregivers in treatment decision making**	
*Client and caregiver level barriers*	• Depression severity; risk to self and/or others; non-attendance; poor engagement; age and/or capacity; stigma; perceptions of paternalism and coerciveness, and experiences of not being involved; concerns about confidentiality
*Clinician level barriers*	• Reluctance to talk about sexual side effects; disagreements between professionals; style and approach of individual clinicians; disorganisation; underestimation of clients’ ability to comprehend information; failure to share information
*Service level barriers*	• Time limitations, including wait lists and high case loads; decisions already being made before clinician sees client (e.g. treatment initiated by another clinician before seeing client); limited treatment options; lack of available services; lack of readily available resources (e.g. fact sheets)
*Broader level barriers*	• Lack of evidence in the area; restriction of government funding to seeing caregivers
*Facilitators*	• Adequate time; culture of the team; treating voluntary clients; having referral options; professional culture; general shift in healthcare culture towards collaborative approaches/informed clients
**How to improve treatment decision making**	• Better information resources (e.g. fact sheets) that are up-to-date, relevant to young people, able to be given to caregivers, readily available, balanced, not overwhelming, available on the Internet and interactive; giving structure to existing conversations (e.g. about treatment); time to think about decisions; being clear about limitations of the service; development of guidelines around involvement and capacity for involvement; training for clinicians; more time

### Participants

Of the twenty-two clinicians who participated, their ages ranged between 25 and 54 years old (mean age 36.9; SD 9.6) and 13 (40.9%) were female. There were ten clinical psychologists (eight working in the public mental health system and two working in private practice); five psychiatrists; four general practitioners (GPs); one mental health nurse; one youth worker; and one youth outreach worker. Clinicians had been working in their respective professions for between one and 30 years (mean 10.7; SD 9); and had been working specifically with young people for between one and 25 years (mean 8.5; SD 7.5).

### Approach to treatment decision making

#### Decision-making model

The vast majority of clinicians reported employing a collaborative approach to decision-making processes (as described by Charles et al. 1999; [28]) either some, or all, of the time. However, they usually advocated for the decision to be made by the client. For example, clinician 12 (female psychiatrist) said about her approach to treatment decision making:

“It’d be collaborative usually, it would all be kind of discussed and the different options would be put forward and then we kind of talk about, you know, the benefits and the disadvantages of the different options and then they would kind of choose a preference”

Ultimately, the decision was seen as belonging to the client. This was described both in terms of psychological therapy and medication:

“How the client uses the therapy is left up to them by default, whether or not the client comes to therapy is their decision and whether or not they engage with other parts of the service, for example group and stuff, that is also their decision in principle” [Clinician 09; female psychologist]

“It always ends with the client, if they don’t want medication or aren’t interested in hearing about it then it’s not really discussed with them, it’s not made important” [Clinician 03; female psychologist]

None of the clinicians reported asking clients or caregivers explicitly about how involved they wanted to be in decision-making processes. Rather, they either didn’t see this as valuable (e.g. because preference for involvement changed too often) or they preferred to gauge it in other ways (e.g. non-verbal cues).

#### Who should weigh up the potential risks and benefits of different treatment options?

During the treatment decision-making processes, clinicians reported presenting treatment options to clients and discussing the potential risks and benefits of treatment options:

“You often present people with the evidence, and that is for the treatment of depression, the combination of medication and therapy, often people find works best, but that’s not to say that you’re not going to get better on just therapy, and then the risks would be the side effects, I guess in that, you know, not every medication works for everyone and sometimes you have to try a different medication and so on” [Clinician 08; female psychologist]

The majority of clinicians believed that a collaborative approach to weighing up the potential risks and benefits of treatment options was most ideal. Other clinicians felt that clients should do it with their support, and still others felt that they themselves should take on the task, commonly citing professional obligations as a reason for this. There was variation in reasons for these responses, however, and for some clinicians there was also a distinction made about the decision-making process and who actually makes the decision. When clinicians spoke about their own role in the ‘weighing up’ process, this varied from supportive (e.g. as an ‘educator’) to directive (e.g. as a ‘driver’).

### Exceptions to decision-making approaches taken by clinicians

Clinicians also described situations in which they would not follow their usual approach. There were four main circumstances described, which all lead to a more paternalistic style: the severity of depressive symptoms experienced by the client and associated decline in functioning; perceived risk levels (i.e. to risk to self or others); perceived client preference for involvement; and the age or developmental stage of the client.

These exceptions were spoken about in terms of a shift in dynamics rather than taking over entirely or employing a strictly paternalistic approach. Clinicians spoke about this shift as if it were on a continuum rather than a categorical change, for example clinician 05 (male psychologist) spoke about a range of issues that would culminate in him adjusting the continuum of involvement: 

“Certainly, with people where their functioning is really deteriorating, their supports are lacking, their engagement is not great, all of these sorts of risk factors, in many ways our level of directiveness (sic) will increase, so as their deterioration worsens, our getting a little bit directive increases”

For clinician 16 (female GP), she felt more adamant that, if there were significant risk issues, “I have to be far more controlling than that… (I) need to ensure that the risk is managed”.

However, when clinicians spoke about a decrease in client involvement, it was usually in terms of increased ‘encouragement’ (e.g. clinician 03; female psychologist), ‘pushing harder’ (e.g. clinician 08; female psychologist) or more strongly ‘recommending’ treatment (e.g. clinician 01; female psychologist). It was also usually about promoting medication rather than psychological therapies or other treatment options.

Several clinicians felt that the client should still have the final decision unless they were being treated involuntarily.

### Reasons for involving clients

Involving clients in decision-making processes (and in making actual decisions) was seen as important to clinicians for several reasons. Several clinicians believed that feeling involved was therapeutic in and of itself, that it could help to facilitate engagement of the client, and that it was the “right thing to do” (e.g. clinician 11; male psychiatrist). Potential negative outcomes of not involving clients as reported by clinicians included non-adherence to medication and disengagement from treatment overall.

### Caregiver involvement

For the majority of clinicians, caregiver involvement was presented as optional and based on the preference of the client.

Clinician 17 (female GP) described how she would usually broach the idea of involving caregivers, using questions such as “are you going to tell your mum” or “would you like me to tell your mum”. Client 18 (female GP) also described how she would ask clients about their preference for caregiver involvement in an ongoing way: 

“(We give) the client first say as to whether they want that (caregiver) in the room… more often than not the younger they are the more they want that person in the room… and at some stage we also double check when they want that person to leave, or I might simply ask them to leave because we’re getting into more delicate questioning”

Clinicians 02 (male psychologist) and 15 (female private psychologist) also believed it was important to convey to the client that the process of involving caregivers was an open one and that they could either be present at, or informed about, any discussion between the clinician and caregiver. Affording clients responsibility for their own care was seen as important for their developmental stage:

“Part of growing up and going through adolescence is individuation and being able to make decisions for yourself and even if those aren’t good decisions (it’s important) that they’re allowed to make those decisions and the process of trial and error” [Clinician 14; female private psychologist]

Within this overarching model of client-directed involvement of caregivers, several clinicians reported making a decision together with the client and then presenting this decision to the caregiver. Clinician 02 (male psychologist) reported that most caregivers were supportive if the decision was explained to them and clinician 09 (female psychologist) would ask them what they thought of the decision. Clinician 10 (female psychiatrist) followed this same process, but said that presenting decisions as “*fait accompli*” to caregivers could be problematic.

Clinicians reported situations in which they would involve caregivers more or less than usual. Reasons for involving caregivers included if there were risk issues with the client, if the client was severely depressed, lacked capacity to make decisions, was younger or less mature. Several clinicians noted that some clients did not have caregivers who were involved, some clients were homeless and others were custodians of the State and this complicated who could act as a caregiver.

In terms of the way in which caregivers could be involved, clinicians commonly cited practical assistance, such as driving clients to appointments; facilitating engagement; taking care of medication in the home; and providing collateral information such as developmental history, current functioning and risk levels, which was valued by clinicians.

Several potential negative outcomes of involving caregivers were spoken about, including disagreements; critical or unhelpful comments; and blurring of boundaries for clinicians not offering family therapy.

Despite seeing difficult or troubled families, positive aspects of involving caregivers were also cited, including the value of communication even when it is difficult; helping to establish that there is a problem; building trust; and supportive caregivers.

### Conceptualising involvement

As the interviews were conducted, a theme not covered by the interview probes was identified in several interviews, that is, how involvement was conceptualised by participants. After conducting the first ten interviews, a question was added to the interview probes: ‘What constitutes true involvement for you?’. As such, the responses in this section are not representative of the group as a whole. Nevertheless, responses are included as they highlight the variation in the concept of involvement and what that might mean for various clients and caregivers.

For clinician 11 (male psychiatrist), true involvement was a step beyond merely agreeing about something (e.g. treatment choice). For him, it was necessary for there to be a “joint understanding” between him and the client, and a prerequisite for this understanding was good engagement. Engagement was important because it meant that the client would listen and trust his judgement, but ultimately he felt that in order to achieve true involvement, “they have to weigh up their own decision making”. Engagement was also a key factor for clinician 15 (female private psychologist), who believed that involvement meant that the client was engaged not only with her but also with the service, had some insight into their own problems and a willingness to be there. That the client had an opinion and felt comfortable enough to openly criticise their experiences of treatment was important to her. Being able to have “mutual agreement and disagreement” was also important for clinician 20 (female mental health nurse):

“Well, I guess it’s having a two way conversation, it’s around allowing the space, the freedom for mutual agreement and disagreement, no, I think that sucks, (or) okay fine, or to have that kind of equal balance conversation I suppose”

Clinician 20 also felt it was important that she didn’t fall into the role of parent or teacher, that she didn’t tell her clients what to do. She believed that in order to involve clients she would offer to have “(an equal) conversation around what fits and what doesn’t” and that she would “respect their decisions” even if that meant that they didn’t want to attend appointments.

Having the client make a decision constituted involvement for clinician 21 (female youth outreach worker), but she also felt that she played a part in this process, and this required her to ask “them what they feel they need… so it’s about giving them the option (of different treatments) and then them picking what it is they need”. This is in line with the bi-directional conversation discussed above by clinicians 15 and 20, and the mutual understanding spoken of by clinician 11.

### Information provision

In terms of the type of information provided to clients, clinicians raised several topics that they typically covered (e.g. depression, therapy, medication); various ways in which they obtained and provided information (e.g. fact sheets, websites); and situations in which they might vary the content or format of information (e.g. with younger clients).

Many clinicians said that they would provide a description of the disorder, either characterising it as a syndrome (e.g. clinician 06; male psychiatrist) or an illness (e.g. clinician 10; female psychiatrist). Several clinicians reported that they would describe depression as common and treatable in order to normalize the experience and promote optimism. Clinician 12 (female psychiatrist) also believed that it was important to describe their impression of the client’s experiences and then “get feedback on whether that’s, if that sounds reasonable (and) from there you’d go into the different treatment options”.

#### Describing potential risks and benefits of treatment options

The potential risks and benefits of medication were spoken about in more detail than the potential outcomes of therapy. In terms of benefits, key messages reported by clinicians included that medication wouldn’t “cure anything” (e.g. clinician 19; male GP) and was not a “magic bullet” (e.g. clinician 05; male psychologist); that it would not work straight away (although the timeframe mentioned by different clinicians differed slightly); that it was likely to help improve depression symptoms faster than psychological therapy (e.g. clinician 07; male psychiatrist); that “the evidence favours a combination of medication and individual therapy” (e.g. clinician 06; male psychiatrist); that it might help to “get them in a bit of a better place to do therapy” (e.g. clinician 02; male psychologist); and that they should still participate in psychological therapy (e.g. clinician 15; female private psychologist). Clinician 07 (male psychiatrist) was “keen not to oversell medication” because the evidence says “they’re not always effective” and that he would feel uneasy if “everything’s gonna be pinned on the (effectiveness of the) medication”. Several clinicians reported informing clients that medication would be effective in approximately 70% of young people (e.g. clinician 06; male psychiatrist). Clinician 14 (female private psychologist) also cited evidence in her information provision.

On the other hand, clinician 04 (male psychologist) said that when he and the treating team presented medication to clients “if anything, there might be more emphasis on the potential benefits than the potential risks (because) we are often coming from the angle of already thinking that it would be useful for the client”. When informing their clients about the potential benefits of medication, clinician 15 (female private psychologist) believed that it was important to get clients to think about “what it’s actually going to provide… is it actually going to help that much”. Similarly, clinician 20 (female mental health nurse) said that she found it helpful to ask clients about their existing knowledge, for example “what do you know about medication, do you know what’s in it, what idea have you got, why do you think it might be helpful”.

The potential risks of taking medication, including side effects, was the most common topic clinicians reported talking with clients about. Clinicians felt that it was important to talk about side effects for various reasons, including that if you didn’t clients would “stop it as soon as they start to get a side effect” (clinician 17; female GP); so that clients could look out for them too (e.g. clinician 05; male psychologist); because it was a clinician’s duty of care (e.g. clinician 13; female psychologist); and so that clients could seek medical attention if they experienced a side effect (e.g. clinician 15; female private psychologist). However, levels of enthusiasm for communicating possible side effects to clients did vary. Clinician 22 (male youth worker), for example, believed that it was “absolutely essential” to let clients know of potential risks because “it’s part of treatment… if I was going there I would want all the information, there’s no difference between me wanting it and a fourteen year old wanting it… they should be given all the information”. Clinician 18 (male GP), on the other hand, felt that there was: 

“…a two edged sword there, it’s a bit like getting people to read the drug inserts in medication, if they read them half the people wouldn’t touch the drugs and I suppose one thing we want to do is to make a reasonable clinical decision here in my own head as to what the issue is and what the best way to approach it is without putting the person off by saying well, look, do you realise… it’s a bit like a surgical consent form, did you realise you could bleed to death, I could lacerate your spleen, or whatever, you don’t want to put them off and particularly in a group that is very quickly disengaged”

In his experience, clinician 02 (male psychologist) said that medical staff might describe side effects, “but they’re not really emphasising (them) a huge amount, I suppose we could explain it more clearly… it’s not really like a clear policy” and that if a client raised any concerns about medication then they would usually provide them with a fact sheet on antidepressant medication. Reasons for minimising the amount of discussion about side effects included that it would take too much time and be a bit “alarming” (e.g. clinician 06; male psychiatrist) for the client.

Although it was not a topic covered specifically by the interview probes, several clinicians raised the issue of the increased risk of suicidality for young people taking selective serotonin reuptake inhibitors (SSRIs). For example, when clinician 01 (female psychologist) was describing the way in which she would communicate information to clients about the potential risks of antidepressant medication, she said that she would tell them about side effects, what to expect, and: 

“…particularly alert them to the risks around agitation and… generally tell them about the fact that there might be a risk of increased suicidal thoughts and agitation and that the young person, if experiencing those things, is to call us straight away and we review it”

Other clinicians did not raise this issue, and although they reported talking to clients about side effects, also said that they would present SSRIs to clients as, for example, “the commonest, it’s the safest, it’s the easiest to prescribe, it’s for the least amount of side effects” (e.g. clinician 17; female GP).

#### Tailoring information

Clinicians raised several situations where they would change the content or delivery of information for clients. Clinicians reported simplifying information for younger clients; clients with lower levels of comprehension or literacy skills; and clients with some type of cognitive impairment. The amount of information provided varied according to clinician; for example, clinician 17 (female GP) said that she gives clients “as much information as I think that they can take in”, whereas clinician 16 (female GP) reported keeping information simple because she believed “we give them too much information… I think providing relevant information enables a decision rather than confusing the matter (with too much information), particularly when people are depressed and their decision-making processes might be impaired”.

#### Information formats

The majority of clinicians said that they just conveyed information orally. Some clinicians felt that written information was useful (e.g. clinician 16, female GP, who believed that “most of what you say in a consultation is forgotten the minute the person walks out”), whereas others did not, such as clinician 18 (male GP), who said that “paper resources I don’t think are particularly useful in this age group, they usually end up out on the street”. Clinician 16 (female GP) also believed that web-based tools helped to engage young people.

In general, clinicians reported using fact sheets from public or not-for-profit organisations and services, such as Orygen, headspace, *beyondblue*, the Black Dog Institute, Reachout, SANE and MIMS handouts. Accessibility of fact sheets varied, with some clinicians reporting that they didn’t hand out fact sheets as often as they felt they should because they didn’t have them in their office (e.g. clinician 10; female psychiatrist) and others reporting that fact sheets were freely available in their office or the waiting room of their service (e.g. clinician 13; female psychologist).

### Negative aspects of client involvement

The majority of interviewees did not report any negative aspects of involving clients, however several clinicians had either experienced downsides of involving clients or could see situations where there might be negative outcomes. Three responses focussed on the potential for the client to disengage or not take up a treatment option that could offer some benefit. For example, client 11 (male psychiatrist) believed that informing clients of the potential risks of medication might make them not want to take it. Ultimately, he felt that the disclosure of such information was important but that there was a need to “balance” what was discussed because he couldn’t tell them about “all of the side effects”. Making sure that the client had understood information was a concern for clinician 09 (female psychologist), who reported “situations where it feels like it hasn’t worked”, where she doubted the “intellectual capacity” of the client and where it had taken a “long time to… try and explain the different options to somebody”. Alongside this, clinician 12 (female psychiatrist) believed that it was a challenge to involve clients when she had to manage a variety of stakeholders: “there’s just so many different people, it’s hard to juggle everybody”, particularly when caregivers were not supportive of clients. Clinician 13 (female psychologist) also spoke of difficulties related to affording autonomy to clients when their caregivers were not supportive of this. Clinician 13 also spoke of the client needing to be ready and mentally well enough to be involved.

Whether or not the client wanted to be involved was raised by clinician 01 (female psychologist), who reported experiences of clients becoming “anxious about the fact that you’re in a position of ‘expert’ and you won’t take up that role”. Still, she felt that once young people experienced involvement then this was generally a “liberating” experience for them.

### Disagreements

Clinicians were asked about any disagreements they had experienced, either with clients or caregivers, and how they dealt with such disagreements.

#### Disagreements with clients

Several clinicians reported not experiencing any disagreements with clients because the approach taken to treatment was based on the preferences of the client. Others said that there were minor disagreements, for example clinician 08 (female psychologist) described facing “little bumps” along the way, so that “you’re constantly negotiating about their treatment”. More significant disagreements were also described, including non-attendance and reluctance to engage in other services (e.g. group programs, drug and alcohol services).

Responses to these disagreements included clinicians trying to understand and “actively explore” (e.g. clinician 02; male psychologist) reasons for decisions made by clients; to present information about treatment options in more detail; to provide more encouragement; to reiterate their position; and to explore any unanswered questions. Ultimately, however, clinicians said that it was up to the client and they could not force clients to agree with them.

#### Disagreements with caregivers

The majority of disagreements related to caregivers either wanting, not wanting, or not being told about medication prescribed to clients. For example, clinician 11 (male psychiatrist) said he thought that: 

“probably everybody’s made mistakes about starting (a medication) and thinking that’s the right thing to do and then having a carer coming (and saying) well why was I not informed about this or whatever”

Ways in which clinicians responded to, or managed, these disagreements included: involving caregivers earlier in the process; further exploring and understanding the perspective of the caregiver; and restating the rationale or justification for their position.

### Barriers and facilitators to involving clients and caregivers in treatment decision making

Clinicians spoke of perceived barriers at four different levels: at a client and caregiver level, a clinician level, a service level, and at a broader level, for example barriers within the community.

#### Client and caregiver level barriers

Several clinicians discussed the experience of depressive symptoms as a barrier to clients being involved in treatment decision making, in that such symptoms impact upon motivation, apathy and engagement in general. The severity of these symptoms was said to vary and therefore have different levels of impact on the ability of clients to be involved.

At the more severe end, the level of risk (e.g. suicidal ideation and behaviours) assigned to clients was something that clinicians considered in terms of the point at which they believed they had to take more control and make decisions for clients. Poor engagement was also seen as a key barrier by several clinicians; as clinician 1 (female psychologist) described it: without involvement “it’s almost impossible to make a decisions, for the young person or for us, to make a decision”.

The age range of the clients was raised as a barrier by psychiatrists, one of whom said that it was an “awkward” age in terms of the legal guidelines around capacity to consent to treatment (clinician 07; male psychiatrist). That the guidelines for capacity were based on age rather than developmental stage was a concern for clinician 12 (female psychiatrist). She felt that in practice she was required to weigh up the autonomy of the client with her own duty of care, but that autonomy took precedent.

Barriers were also raised relating to preconceived perceptions held by clients about mental health services. These included stigma about mental disorders and mental health services, perceptions of paternalism and coerciveness, and experiences of not being involved that have led clients to not expect to be involved. Concerns about confidentiality were also raised more broadly as a barrier to clients being involved in treatment decision-making and disclosing information in general. Only one barrier was raised with specific reference to caregivers, which was that parental conflict could preclude the involvement of the client (clinician 11; male psychiatrist).

#### Clinician level barriers

There were fewer barriers reported by clinicians in terms of their own behaviours and there was less consistency across clinicians than seen in client and caregiver barriers. Clinician-level barriers included a reluctance to talk about sexual side effects; disagreements between professionals (e.g. between case managers and medical staff); the differing styles and approaches of individual clinicians; disorganisation (e.g. not having fact sheets printed out and ready to be given to clients); an underestimation by clinicians in general of clients’ ability to comprehend information; the presentation of information (i.e. that it could influence decisions made by clients and therefore could potentially act as a barrier to true involvement); and lastly, failure to share information between colleagues (e.g. not reading clinical notes).

#### Service level barriers

Clinicians commonly reported two service level barriers: time limitations and the fact that some decisions were already made before they saw clients. Time limitations were discussed in relation to the length of appointments (e.g. not enough time to discuss all of the potential risks and benefits of treatment options); the number of government subsidised appointments with private psychologists; and the duration of care restrictions for clients in the public health system, particularly for clients who have already had a past episode of care.

Related concerns included having a waiting list for their service (and therefore if a client did not engage then they were discharged), and having high caseloads resulting in time pressures and less frequent appointments.

Clinicians also believed that it was difficult to involve their clients in some decisions because these decisions had already been made before seeing them. This involved decisions having been made at the same service but by other clinicians (e.g. entry or assessment teams, acute services and inpatient units) and also decisions having been made by other services (e.g. in general practice).

#### Facilitators

When discussing barriers to involving clients in treatment decision-making, several clinicians also volunteered facilitators, or factors that make it possible, to involve clients in such decision-making. Having adequate time was the most common response. The culture of the team within which clinicians worked was also a facilitator, for example that the clinic supported collaboration with clients (clinician 06; male psychiatrist); being able to raise issues in clinical review settings, and not having concerns about the client trivialised, and therefore feeling more supported to involve clients (clinician 21; female youth outreach worker); not having to see clients being treated involuntarily and having referral options if treatment is not working with her (clinician 20; female mental health nurse); working in a profession where clients tend to be “a bit more open” than, for example, with medical doctors (clinician 22; male youth worker); and what clinician 10 (female psychiatrist) saw as a general shift in healthcare culture towards a more collaborative approach with more informed clients.

### How to improve treatment decision making

The most common response from clinicians when asked what they thought would improve treatment decision making, was to have better information resources (e.g. fact sheets). Clinicians valued having fact sheets that were up-to-date, relevant to young people, able to be given to caregivers and readily available. Suggestions for fact sheets included that they be balanced, unbiased (e.g. “not driven by litigation and drug companies”; clinician 16, female GP), not overwhelming, to have simple messages, to be available on the Internet, and to be interactive; “anything you can do interactively, like getting (clients) to write things in and you write things in is good” (clinician 10, female psychiatrist).

Giving structure to existing conversations (e.g. of doing or not doing treatment) was suggested by clinician 02: “formally going through what might be the pros and cons would be helpful”. Having the information available in written format was valued in order to allow clients the time to process the information. Information was also desired for ongoing treatment decision-making, for example clinician 09 (female psychologist) suggested that fact sheets and a protocol for “any kind of change in treatment… or a change in medication… that people are given a fact sheet (and told) ‘go away and think about this for a week’”. Having time to think about decisions was also important to clinician 04 (male psychologist), who believed that it was necessary to have “more checking that the young person’s okay with it… giving it a bit longer to seep in” and to clinician 11 (male psychiatrist) who felt that “people only take in twenty five per cent of what you’re saying anyway” and that by providing “useful educational stuff” the client would have “something to go away with and read and… a green light to come back and say I’ve experienced this (side effect)”.

Being upfront about what the service could and could not provide (e.g. continuing care but not an outreach service) was a concern for clinician 05 (male psychologist), and giving clients realistic expectations in general was endorsed by several clinicians.

Examples of resources (other than fact sheets) that were suggested include guidelines, specifically formal guidelines about involvement and the capacity for involvement, and training for clinicians, for example “in the soft engagement side of things with kids” (clinician 22, youth worker). Lastly, time was seen as a key factor in how to improve treatment decision-making. For example, clinician 08 (female psychologist) said “we are always under time constraints to get people in and get them out again”. Despite all of these recommendations for ways in which to improve the decision-making process, when clinicians were asked if they ever wished the process was different, they all responded by saying ‘no’.

## Discussion

Clinicians endorsed, and reported employing in the majority of cases, a collaborative approach to treatment decision making for young people diagnosed with MDD. In the process of making decisions many clinicians felt that it was an ideal situation to have the client, caregiver and clinician weigh up the potential risks and benefits of different treatment options. Ultimately, however, it was felt that the client themselves had the final say when it came to accepting or declining both psychological therapies and antidepressant medication. This highlights not only the differentiation made by clinicians about the process of decision making and who actually makes the decision [29], but also the similarity in beliefs of clinicians when compared with the variety of perspectives presented by clients, where there was less agreement (as discussed in previously reported data [25]). The variation seen in descriptions of involvement also highlights that roles taken on by clients, caregivers and clinicians are more complex and variable than the categories of autonomous (whereby the client access information from the clinician and then decides themselves), shared (whereby the client and clinician share information and work together to make a decision) and paternalistic styles (whereby the clinician asks the client minimal questions and then makes a decision for them) [30]. In order to maximise the chance that the young person receives their preferred level of involvement, and type of involvement, clients should be asked explicitly about the type and level of involvement they prefer when it comes to treatment decision making. In studies assessing observed rates of SDM behaviours both in mental health (e.g. [31-34]) and in general [35], very low levels of this particular behaviour (i.e. asking clients about their preferred level of involvement) have been reported. Given that preferred level of involvement is likely to change over time [25], this important step in the SDM process will likely need revisiting in the context of chronic disorders and/or ongoing care. Further, preference for involvement should be discussed once the client grasps the concepts of preference-sensitive decisions and how and why they might be involved [36].

Many clinicians felt that considering the values and preferences of clients and their caregivers was important, however values and preferences were not asked about routinely; instead, they were discussed and addressed as the need arose (e.g. if raised by the client). Client preference for involvement was also not routinely asked about. Both the routine consideration of individual characteristics, values and preferences, and explicitly asking about preference for involvement are key steps in models of SDM, the dominant framework for collaborative treatment decision making [28,29,37]. Clinicians’ accounts here are in line with studies investigating levels of SDM behaviours in recorded clinical interactions, which demonstrate relatively low levels of SDM [31-33]. The adoption of these SDM behaviours is critical if clinicians are to account for the variation in client preference for involvement and the different ways in which clients conceptualise involvement, as shown in our previous work [38].

Consideration also needs to be given to the ways that clients *can* be involved when their preferred level of involvement is not afforded to them. In the current study, several circumstances or situations were described in which clinicians would limit the amount of involvement afforded to clients. In line with experiences reported by clients in previous data [25], these situations included clients’ age and when clients had more severe depressive symptoms and higher levels of risk. Clinicians added to this their own assumption of client preference for involvement. One of the few negative aspects related to involving clients, reported by some clinicians, was the possibility of overwhelming the client when they were unwell. Given that clinicians did not report asking explicitly about client preference for involvement, it is possible that discrepancies may arise between perceived and actual client preference for involvement. For example, in our previous work, some clients were accepting of having reduced levels of involvement whereas others were not [25]. Even when clinicians feel that full involvement is not possible, it may be beneficial to maintain involvement of clients in terms of upholding rapport throughout these compromised situations. In doing so, affording clients control over some aspects of their treatment (e.g. type of medication to take or psychological therapies to engage in) may compensate for being excluded from larger decisions (e.g. being treated as an inpatient rather than in the community).

Despite this variation in how clients were actually involved in the decision-making process, clinicians felt that using a collaborative approach with clients was important. They believed that involvement was therapeutic in itself, that it promoted autonomy and that it was important for clients developmentally. They also predicted that if they failed to employ a collaborative approach and involve clients in the treatment decision-making process, that clients would be non-adherent and/or disengage. Again, this is supported by previously reported client data, where young people reported not taking medication and disengaging from services after failing to be involved by clinicians [25]. When it came to defining this involvement, clinicians focused on aspects of the client-clinician relationship such as engagement, and having the client feel comfortable enough to explicitly decline treatment options. In previously reported work, clients also felt that relationship-related factors such as engagement and trust were critical [25]. Taken together, these responses support the notion that involvement should be considered not only as specific actions (e.g. sharing information, talking about this information), but also in terms of the feelings that each person has for the others involved in the treatment decision-making processes [39,40]. This might also be an important part of why clinicians place importance on involvement for outcomes related to client engagement and adherence.

Along with talking about the importance of involving clients, many clinicians also believed that caregiver involvement was ideal. Ultimately, however, they said that it was optional and based on the preferences of the client. Clinicians said that it was best for caregivers to agree with decisions, but that they were not necessarily decision makers. Rather, they were seen as important contributors to practical aspects, such as looking after medication. This is consistent with the experiences that caregivers reported in our earlier work [25], but is contrary to their reported desire to be more involved than this. Clients on the other hand reported being happy with caregivers playing a supportive role rather than being involved directly in treatment decision making [25]. Such a role may not match the desired level of involvement and definition of involvement as viewed by caregivers. When disagreements arose between clinicians and caregivers, or between clinicians and clients, the main approach reported was to explore reasons behind disagreements (e.g. the reasons for a client refusing medication) and restate their rationale and justification for their own position. Again, clinicians felt that ultimately the final decision rested with clients and that they could not force voluntary clients to engage in treatment, but there is potential for this friction to act as a barrier to a more collaborative approach.

Indeed, many clinicians saw engaging clients as a key barrier to their involvement, saying that they could not involve them if they were not attending sessions and willing to be seen by the service. When asked to identify barriers to involving clients or caregivers in treatment decision-making processes, clinicians detailed a number of issues relating to clients, clinicians, service settings and broader factors. There were similarities between the barriers reported by clinicians in the current study and those described by clients in previously reported data (e.g. accessing services; clinician style), however caregivers in this past study focussed more on service-level barriers such as age-related confidentiality policies [25]. Barriers reported by clinicians in the current study were somewhat consistent with those identified across a variety of settings [41]. In particular, barriers relating to clinician perceptions about client characteristics and preferences, which may lead to assumptions that certain client should not be involved.

When asked about how treatment decision-making processes could be improved, clinician showed a strong desire for more informative resources for both clients and caregivers. Fact sheets that were up to date, relevant to young people, able to be given to caregivers, readily available, balanced, web based and interactive were valued. Clinicians were asked about the type and amount of information they provided clients, and the variation in their Reponses demonstrates a key gap in the decision making process. Given that clinicians also described limiting involvement for some clients (as described above), it seems important to have information available to all clients, who can then access it if they want to. Clinicians’ desire for more informative resources was also seen in interviews with clients and caregivers [25], who did not always receive adequate information and had to look elsewhere for it. Informative tools such as decision aids [42] may be useful for this population, satisfying the needs of clients, caregivers and clinicians. This again lends support to the use of SDM, which decision aids can help to facilitate.

The current study has several limitations, including the fact that participants from Orygen Youth Health and headspace may have been more likely to prefer or report collaborative approaches to treatment decision making because this is the culture and policy of these services. Recruitment was extended to clinicians from other services, which may or may not have similar organisational cultures and policies, to try and capture a broader range of experiences and beliefs. Additionally, participants were from a range of professional backgrounds and were only able to discuss decisions related to the type of treatment they provide. For example, non-medical clinicians are limited in how much they can discuss decisions about medication. Although we tried to include a broad range of professional backgrounds, in some cases only a small number of participants were included from certain professions (e.g. youth work). This limits the generalizability of the results across professions, however we would note that the spread of professional backgrounds included in the study is similar to the ratios seen at the two main services we recruited from.

Another limitation is that participants were discussing instances where the other people present (e.g. clients and caregivers they saw) were not interviewed. We relied on accounts of experiences and beliefs, which are vulnerable to biases (e.g. social desirability bias). An alternative approach, for example, may have been to interview a client, caregiver and clinician about the same instance of treatment decision making. In doing so, more direct comparisons could be made about the similarities or variations in accounts. However, it is anticipated that recruitment of such a sample would have been more difficult and resulted in a smaller numbers of participants. We could also have recorded clinical encounters where treatment decision making occurred to measure the level of SDM behaviours. Instead, the approach taken in the current study was to sacrifice this triangulation or recording of actual instances for a broader range of perspectives about a larger number of situations. Participants were recalling events they experienced over several years, and may have been more likely to recall experiences where they had acted more similarly to their ideal model of decision making (e.g. collaborative). In line with our approach, rather than attest to the accuracy of accounts, this study has instead sought to consider variations in experiences of involvement. Also, consideration of beliefs from a broader range of participants has been possible. This was important given the aim of the study was to obtain rich descriptions of a variety of experiences and beliefs, something that is lacking in this area of research.

## Conclusions

Overall, previously reported interview data from clients and caregivers [25] and the current interview data from clinicians, has demonstrated that some type of collaborative approach to treatment decision making for young people diagnosed with MDD is seen as the ideal model. An emphasis was placed on the decision-making processes [29] and having high quality information about treatment options that is evidence-based, encourages reflection on personal characteristics, values and preferences, and is freely available is a gap identified in these data. Filling this gap may allow for a base level of involvement that can be afforded to all clients, and used in a flexible way dependent on their preferences for information and involvement in decision making. Asking all clients explicitly about their preference for involvement could also help to clarify any discrepancies between perceived and actual preference for involvement in decision-making processes.

Having the opportunity to share some involvement where possible may lead to higher levels of satisfaction and engagement for clients who would otherwise be denied their preferred level of involvement. The preferred model by the majority of all clinicians, and clients and caregivers in previously reported data [25], was most in line with SDM. Given that SDM is very often facilitated with the use of informative, evidence-based decision making tools called decision aids [42], this approach also has the potential to fulfil the desire of all participant groups for more informative resources.

This study is the first to specifically consider the experiences and beliefs about treatment decision making for young people diagnosed with MDD. This study fills a gap in the knowledge about the context in which young people diagnosed with MDD find themselves making treatment decisions. Importantly, this study provides empirical data that can contribute to the development of ‘youth shared decision making’ frameworks (e.g. (203)) and how clinical guidelines that advocate for the inclusion of young people in treatment decision making (e.g. (26, 30)) might be practically realised.

### Declaration

This study is presented in line with the RATS guidelines for the reporting of qualitative research [43].

## Competing interests

The authors have no competing interests to declare.

## Authors’ contributions

MS conceived the project, conducted the interviews, analysed the data and drafted the manuscript under the supervision of SH and AJ. SH and AJ were also involved in subsequent redrafts of the manuscript. All authors read and approved the final manuscript.

## Authors’ information

MS is a Research Fellow, SH is a Senior Research Fellow, and at the time of this work AJ was a Professorial Fellow at Orygen Youth Health Research Centre, Centre for Youth Mental Health, The University of Melbourne. AJ is now a Professorial Fellow at the Population Mental Health Group, Melbourne School of Population Health, The University of Melbourne. This work was undertaken by MS in order to fulfil the requirements of a PhD.

## Pre-publication history

The pre-publication history for this paper can be accessed here:

http://www.biomedcentral.com/1471-244X/13/335/prepub
